# Remifentanil versus Propofol/Fentanyl Combination in Procedural Sedation for Dislocated Shoulder Reduction; a Clinical Trial

**Published:** 2019-01-25

**Authors:** Vahid Monsef Kasmaee, Seyed Mahdi Zia Zibari, Marjan Aghajani Nargesi

**Affiliations:** 1 Guilan Road Trauma Research Center, School of Health, Guilan University of Medical Sciences, Rasht, Iran. E-mail:vmonsef@yahoo.com; 2 Department of Emergency Medicine, School of Medicine, Guilan University of Medical Sciences, Rasht, Iran. E-mail: Smzz5859@hotmail.com

**Keywords:** Propofol, remifentanil, fentanyl, shoulder dislocation

## Abstract

**Introduction:**

Procedural sedation and analgesia (PSA) is a fundamental skill for every emergency physician. This study aimed to compare the PSA characteristics of remifentanil with propofol/fentanyl combination.

**Methods:**

In this double-blind randomized clinical trial, the procedural characteristics and number of failures, as well as adverse events were compared between groups treated with either remifentanil or propofol/fentanyl combination consisting of 15-60 year old patients referring to emergency department following acute anterior shoulder dislocation.

**Results:**

64 patients were randomly assigned to either remifentanil (32 cases) or propofol/fentanyl, (32 cases) groups. The two groups were similar regarding mean age, sex, and pain severity at the time of presentation to ED. The two regimens had the same efficiency regarding pain management (100% success rate). 22 (68.8%) cases in remifentanil group and 4 (12.5%) cases in propofol/fentanyl group had failed in muscle relaxation (p < 0.001). In the group receiving remifentanil, onset of action (p = 0.043) and recovery time (p < 0.001) were significantly shorter. 10 (31.3%) cases in remifentanil group and 11 (34.4%) cases in the other group experienced adverse events (p =0.790). There was a significant difference between groups regarding the type of adverse events (p = 0.003).

**Conclusion:**

Compared to propofol/fentanyl combination, remifentanil has equal efficiency in pain management, lower success rate in muscle relaxation, significantly higher frequency of apnea, and shorter onset of action and recovery times in PSA for reduction of anterior shoulder dislocation.

## Introduction :

Shoulder dislocation rate is around 23.9 for 100,000 population in the United States (1). Analgesia and muscle relaxation are often induced for facilitating reduction. Various combinations and types of drugs are used for this propose. In many institutions, the preferred method for providing adequate analgesia involves tranquilization using a combination of benzodiazepines and narcotics (2-4). 

Propofol is a sedative drug with a dose-dependent effect used for general anesthesia. It has no analgesic properties, so it should not be used as the sole drug in moderately painful procedures. It is suitable for emergency treatment because of its rapid onset and anti-nausea/vomiting effects (5, 6). 

Remifentanil is a relatively new synthetic opioid with properties similar to fentanyl (2, 18). However, there is still controversy regarding the effectiveness of using remifentanil alone for procedural sedation and analgesia (PSA) (7, 8).

Dunn et al. have introduced remifentanil as an excellent analgesic and sedative agent in PSA for reduction of anterior shoulder dislocation (9). It has been reported that remifentanil has lower failure rates and higher patient satisfaction compared with fentanyl in this regard (2). 

Searching for the best choice for PSA induction resulting in higher physician and patient satisfaction and fewer adverse effects, this study was designed aiming to compare the PSA characteristics of remifentanil with propofol/fentanyl combination in reduction of anterior shoulder dislocation in emergency department.

## Methods:

***Study design and setting***

The present study is a double-blind randomized clinical trial performed on patients with anterior shoulder dislocation presenting to Poursina Hospital, Rasht, Iran, from January to August 2017, to compare the PSA characteristics of remifentanil with propofol/fentanyl combination. The study protocol was approved by ethics committee of Guilan University of Medical Sciences under the number IR.GUMS.REC.1396.271 and registered on Iranian registry of clinical trials under the number IRCT20110818007369N6. Researchers adhered to the principles of Helsinki ethical recommendations and confidentiality of patients’ information throughout the study period.

***Participants***

Patients with acute anterior shoulder dislocation aged between 15-60 years were included in this study. Those with fracture-dislocation of the shoulder joint and history of surgery, except for patients with Hill-Sachs lesions, as well as patients with decreased consciousness and unstable hemodynamic status, hypotension (SBP <90), history of heart disease, and allergy to soy and eggs were excluded. 

***Procedure***

After careful history taking and physical examination, eligible patients underwent continuous cardiac, respiratory, blood pressure, and consciousness monitoring throughout the procedure. Patients were randomly assigned to either remifentanil or propofol/fentanyl group using block randomization method. For the first group, a combination of propofol (1 mg/kg, produced by DarooPakhsh Company, Tehran, Iran) + fentanyl (1 μg/kg, produced by DarooPakhsh Company, Tehran, Iran) and for the second group, remifentanil (1 μg/kg, produced by Hameln, Germany) were administered, intravenously. 0.5 mg/kg propofol for propofol/fentanyl group and 0.5 μg/kg remifentanil for remifentanil group were considered as rescue dose. In case of problem in muscle relaxation, 0.5 mg/kg propofol was administered regardless of the group. Patients, the physician, and the statistical analyzer were blinded to the type of drug injected. All reductions were performed by emergency medicine residents and trained nurses in charge of patients using traction counter-traction method.

***Data gathering***

A checklist containing demographic data (age, sex); pain severity before, during, and after reduction; onset of drug action; time to recovery; muscle relaxation; need for rescue doses; and adverse events was filled out for each participant by a senior emergency medicine resident under supervision of an emergency medicine specialist. Severity of pain was measured via visual analogue scale (VAS). In this study, success was defined as ≥ 3 points decrease in pain severity on VAS and recovery time was defined as time interval between dislocation reduction and complete orientation of patients.

***Outcomes***

Success rates in pain management and muscle relaxation were considered as main outcomes and adverse events as well as onset of action and recovery times as secondary outcomes.

***Statistical analysis***

Data were analyzed using SPSS software version 18 and with intention to treat analysis method. Independent t-test, chi-square and Fisher’s exact tests were used for comparisons. Normality of the data was measured by the KS test. In this study, p = 0.05 was considered as significance level. Findings were presented as mean ± standard deviation or frequency and percentage.

## Results:

***Baseline characteristics of patients***

64 patients were randomly divided into 2 groups of remifentanil (32 cases) and propofol/fentanyl, (32 cases). Mean age of patients was 34.28 ± 10.84 years in remifentanil group and 35.43 ± 14.25 years in the propofol/fentanyl group (p = 0.716). Table 1 compares the baseline characteristics of the two groups. The groups were similar regarding mean age, sex, and pain severity on presentation to ED.

***Outcomes***

**Figure 1:**
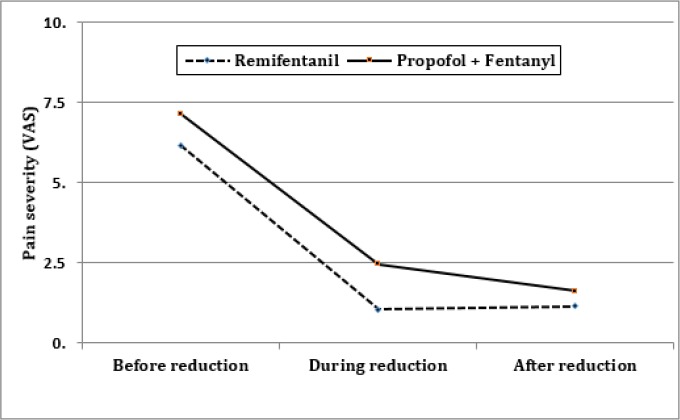
Comparison of pain severity between the groups at different studied times

Table 2 compares the studied outcomes between groups. As table 2 and figure 1 show, although the severity of pain during and 20 minutes after reduction was statistically different between the two groups, it was not clinically important. In other words, the two regimens have similar effectiveness in pain management (100% success rate). 22 (68.8%) cases in remifentanil group and 4 (12.5%) cases in propofol/fentanyl group had failure in muscle relaxation and needed additional dose/s of propofol for muscle relaxation (p < 0.001). Remifentanil receiving group had a significantly lower onset of action (p = 0.043) and recovery time (p < 0.001). 10 (31.3%) cases in remifentanil group and 11 (34.4%) cases in the other group experienced adverse events (p =0.790). There was a significant difference between groups regarding the type of adverse events (table2; p = 0.003).

## Discussion:

Based on the findings of the present study, compared to propofol/fentanyl combination, remifentanil is equally effective in pain management, but has lower success rate in muscle relaxation, significantly higher frequency of apnea, and shorter onset of action and recovery times in PSA for reduction of anterior shoulder dislocation.

**Table 1:** Baseline characteristics of the two studied group

**Variable**	**Remifentanil**	**Propofol/fentanyl **	**P**
**Sex **			
Male	11 (34.4)	5 (15.6)	0.083
Female	21 (65.6)	27 (84.4)	
**Age (year)**			
Less than 30	11 (34.4)	14 (43.8)	0.674
31-40	12 (37.5)	9 (28.1)	
41-60	9 (28.1)	9 (28.1)	
**Pain severity (VAS)**			
On presentation to ED	6.2 ± 1.9	7.3 ± 3.2	0.110

Data are presented as mean ± standard deviation or frequency (%) VAS: visual analogue scale.

**Table 2:** Comparison of studied outcomes between groups

**Variable**	**Remifentanil**	**Propofol/fentanyl **	**P**
**Pain severity (VAS)**			
During reduction	1.06 ± 0.35	2.40 ± 1.34	< 0.001
20 minutes after reduction	1.15 ± 0.36	1.59 ± 0.79	< 0.001
**Success rate **			
Pain management	32 (100.0)	32 (100.0)	NA
Muscle relaxation	10 (31.3)	28 (87.5)	< 0.001
**Adverse events **			
Agitation	0 (0.0)	8 (25.00)	0.003
Apnea	10 (31.30)	3 (9.40)	
**Time (minutes)**			
Onset of action	1.29 ± 0.48	1.96 ± 1.77	0.043
Recovery	2.06 ± 1.00	5.43 ± 4.93	< 0.001

Data are presented as mean ± standard deviation or frequency (%) VAS: visual analogue scale.

These findings are similar to studies by Dunn et al., Phillips, Swann, Sacchetti, and Cok OY et al., (9-12). In the study by Gharavifard et al., which was performed comparing groups receiving either midazolam/fentanyl or remifentanil, the failure rate in reduction was 15 (31.3%) cases in the first group and 1 (2.1%) case in the second one (2). 

Rai et al., studied remifentanil versus propofol for fiber optic intubation; their report indicated a shorter endoscopy and intubation time for the Remifentanil group (13). Maltepe and colleagues found that recovery after propofol/remifentanil administration was faster than the combination of propofol/fentanyl (14). Recovery periods in Ozkun et al. study were shorter than the recovery period in similar studies performed on propofol combinations (15-17). 

In the present study, eight cases of agitation and 3 cases of apnea in the propofol/fentanyl group, as well as 10 cases of apnea in the remifentanil group were detected. Although remifentanil provided a faster recovery, it had considerable respiratory side effects. In the study by Ozkun et al., remifentanil caused the most respiratory complications, yet these effects were transient and none of them required mechanical ventilation. The use of remifentanil in combination with midazolam in pediatric PSA has led to high and unacceptable levels of hypoxemia (18).

Similar to findings of the present study, in Ozkun et al. trial none of the patients experienced nausea and vomiting after anesthesia (15). This may be due to the anti-inflammatory effects of propofol or lower doses of opioids used in these studies. 

It seems that use of remifentanil resulted in shorter recovery time but higher failure rate in comparison with propofol/fentanyl combination in anterior shoulder dislocation. The analgesic effects were approximately the same in both groups. Although remifentanil has a shorter recovery time, the considerable respiratory side effects and the intensity of muscle stiffness after the injection, made it unsuitable for being used as the sole drug for reduction of anterior shoulder dislocation.

Overall, it should be stated that for choosing the proper drug for a procedure it should be considered whether the main goal is pain control or analgesia or a combination of both. It seems that for a procedure such as shoulder dislocation, which needs both analgesia and sedation for facilitating the reduction process, using remifentanil as the sole drug with the dose used in the present study is not a good choice. However, it should be noted that some opioid drugs such as fentanyl show sedative effects in higher doses.

## Conclusion:

Based on the findings of the present study, compared to propofol/fentanyl combination, remifentanil is equally effective in pain management, but has lower success rate in muscle relaxation, significantly higher frequency of apnea, and shorter onset of action and recovery times in PSA for reduction of anterior shoulder dislocation.
